# Biomechanical study of Tile C3 pelvic fracture fixation using an anterior internal system combined with sacroiliac screws

**DOI:** 10.1186/s13018-021-02348-y

**Published:** 2021-03-27

**Authors:** Lin Liu, Donggui Zeng, Shicai Fan, Yongxing Peng, Hui Song, Dadi Jin, Letian Zeng

**Affiliations:** 1 Orthopedic Trauma, University of Chinese Academy of Sciences Shenzhen Hospital, Guangdong Shenzhen, People’s Republic of China; 2 The Third Affiliated Hospital, Southern Medical University, Guangzhou, Guangdong People’s Republic of China

**Keywords:** Internal fixation system, Pelvic fracture, Tile C3, Minimally-invasive, Biomechanics

## Abstract

**Background:**

How to perform minimally-invasive surgery on Tile C pelvic fractures is very difficult, and it is also a hot topic in orthopedic trauma research. We applied minimally-invasive treatment using an anterior internal fixator combined with sacroiliac screws.

**Objectives:**

To compare the biomechanical properties of different fixation models in pelvic facture specimens, using an internal fixation system or a steel plate combined with sacroiliac screws.

**Methods:**

Sixteen fresh adult cadaver pelvic specimens were randomly separated into four groups named A, B, C, and D. The four groups were respectively stabilized using a two-screwed, three-screwed, or four-screwed anterior internal fixator or a steel plate with sacroiliac screws. All models were tested in both standing and sitting positions. Vertical loads of 600 N were applied increasingly. Shifts of bilateral sacroiliac joints and pubis rupture were measured.

**Results:**

The shifts in sacroiliac joints and pubis rupture in the standing position were all less than 3.5 mm, and the shifts in the sitting position were all less than 1 mm. In the standing position, the results of shifts in the sacroiliac joints were group C < group D < group B < group A. For comparisons between A:B and C:D, *P* > 0.05. For comparisons between A, B:C, and D, *P* < 0.05. The results of shifts in pubis ruptures were group D < group C < group B < group A. In the comparison between C:D, *P* > 0.05; for comparisons between A:B, A:C, A:D, B:C, and B:D, *P* < 0.05. In the sitting posture, the results of shifts in the sacroiliac joints were group C < group D < group B < group A, and the shifts in the pubis ruptures were group D < group C < roup B < group A. For comparison between C:D, *P* > 0.05. For comparisons between A:B, A:C, A:D, B:C, and B:D, *P* < 0.05.

**Conclusion:**

Use of an anterior internal fixator combined with sacroiliac screws effectively stabilized Tile C3 pelvic fractures. The stability of specimens increased as the number of screws in the internal fixator increased.

## Introduction

With the development of minimally-invasive techniques, there is increasing interest in treating pelvic fractures with these methods. Currently, the most common surgical technique to fix a vertically-unstable posterior pelvic ring fracture is to use sacroiliac screws for closed reduction. For the treatment of anterior pelvic ring fractures, some researchers have reported that the use of subcutaneous screws and rods for internal fixation has good efficacy. This method is known as a subcutaneous internal fixator [1, 2] or an anterior subcutaneous pelvic fixator (INFIX) [3, 4].

**Fig. 1:**
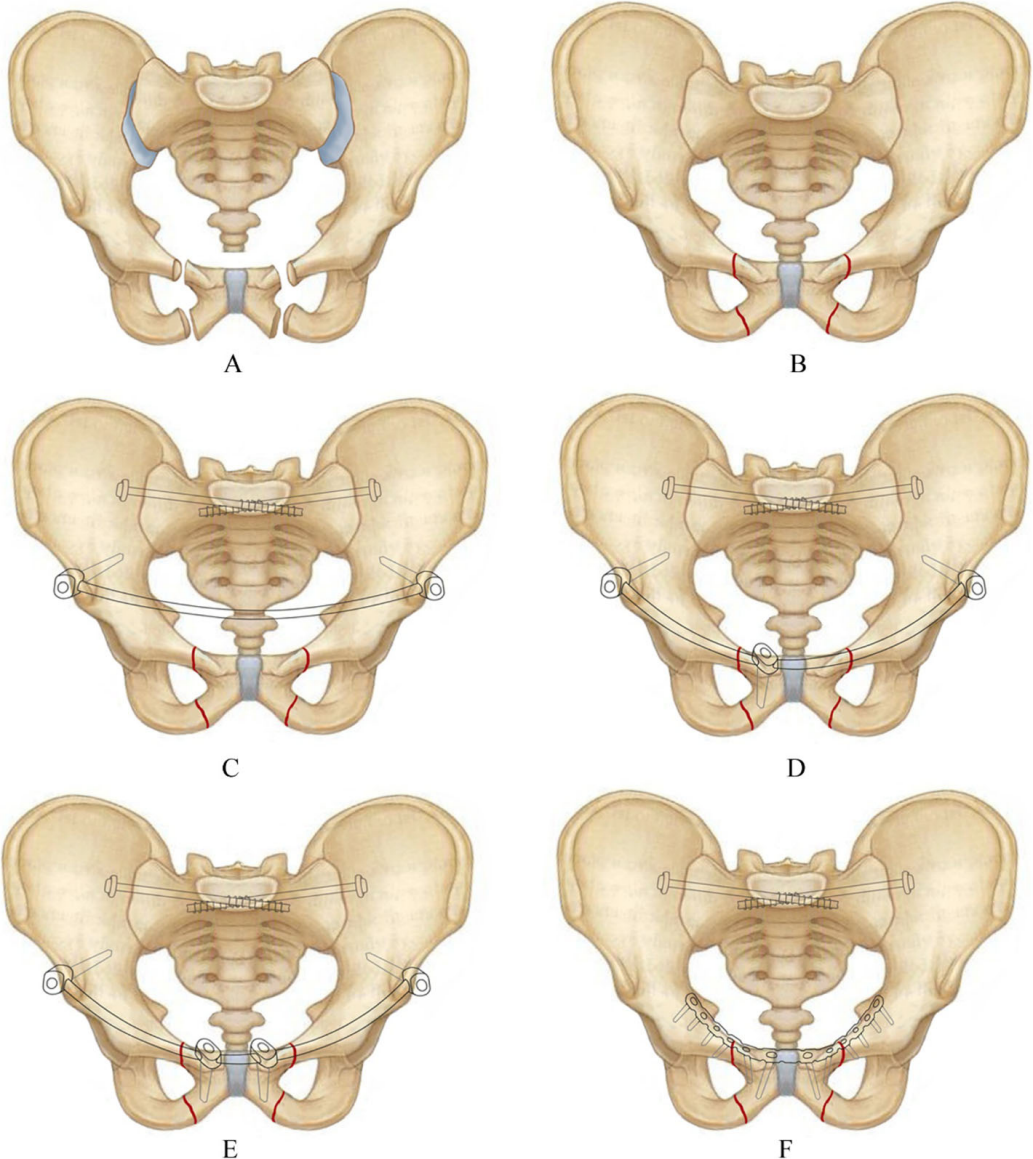
Facture models and fixation methods. **a** Tile C3 pelvic fracture. **b** Reduction of fracture. **c** Fixation model using 2 screws anterior internal fixation system combined with sacroiliac screws. **d** Fixation model using 3 screws anterior internal fixation system combined with sacroiliac screws. **e** Fixation model using 4 screws anterior internal fixation system combined with sacroiliac screws. **f** Fixation model using plate combined with sacroiliac screws

Tile C3 is considered to be the most severe pelvic fracture type because the anterior and posterior pelvic rings are both severely unstable and such fractures are usually combined with other organ injuries. Treating Tile C3 pelvic fractures using early minimally-invasive techniques still remains one of the big challenges for surgeons. Therefore, we have suggested the innovative approach of applying minimally-invasive treatment to Tile C3 pelvic fractures by using an anterior internal fixator combined with sacroiliac screws. To provide effective theoretical support for such a clinical application, we tested the biomechanical properties of fixation models of Tile C3 fractures (Fig. 1) using the internal fixator system and a steel plate with sacroiliac screws.

## Materials and methods

### Preparation and sampling of specimens

**Fig. 2:**
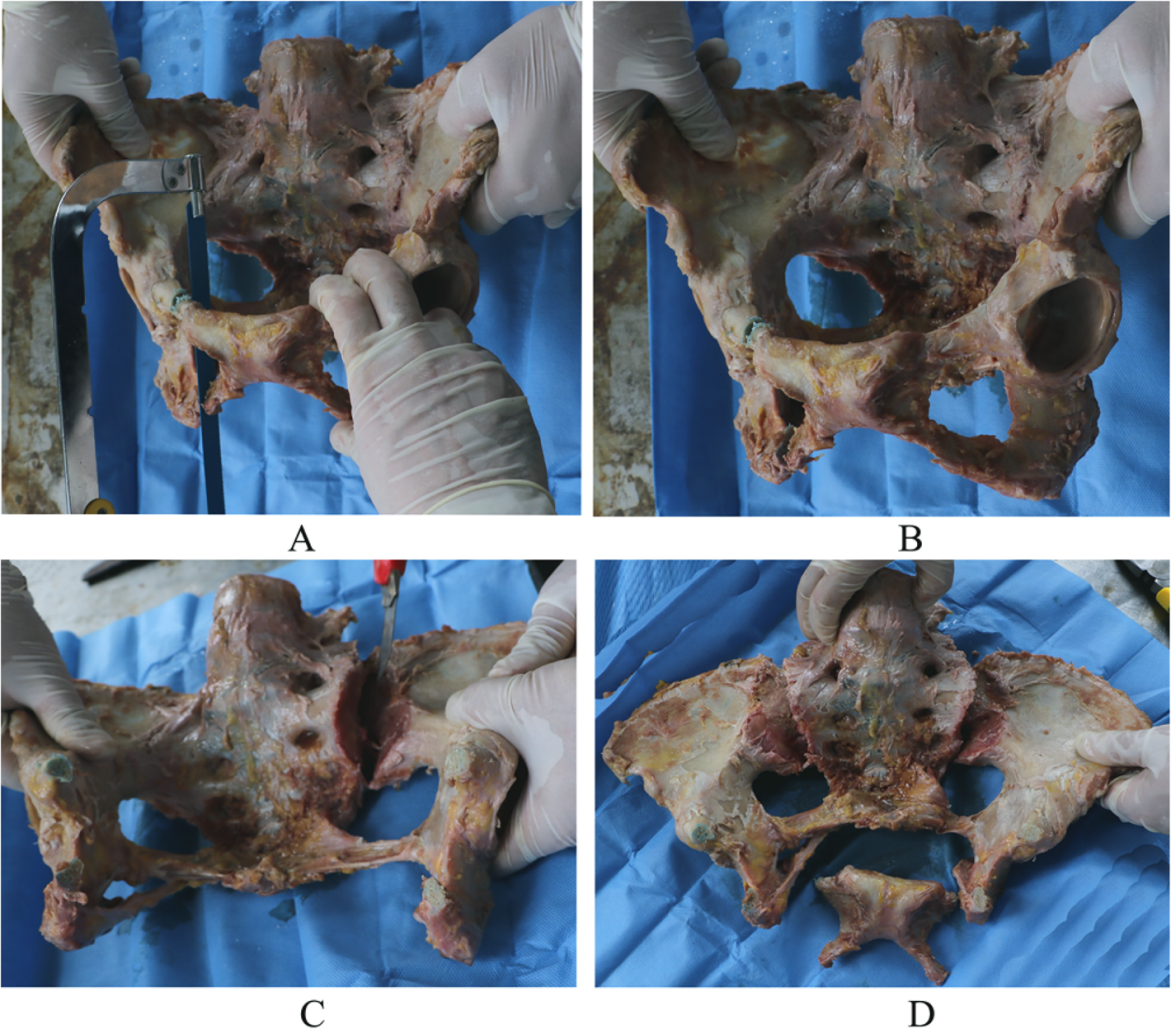
Tile C3 pelvic fracture preparation. **a**, **b** Pubic fracture. **c** Sacroiliac joint dislocation. **d** Tile C3 pelvic fracture model.

Sixteen fresh ice-cold adult pelvises (10 males, six females, aged 25–66 years, average age 43.5 years) provided by the Department of Anatomy in Southern Medical University were used for the biomechanical tests. All the specimens were observed both by the naked eye and by CT examination. Any specimens with a fracture, tumor, malformation, or marked osteoporosis were all excluded. Each of the specimens was a complete pelvis including the L5 vertebral column. The anterior sacroiliac ligament, long dorsal sacroiliac ligament, short posterior sacroiliac ligament, interosseous sacroiliac ligament, sacrospinous ligament, and sacrotuberous ligament were all maintained on the specimens. The specimens also had the complete symphysis pubis including the superior pubic ligament, arcuate ligament, and anterior pubic ligament. All the specimens were labeled and randomly grouped into group A, B, C, or D with four specimens in each group. In groups A, B, and C, a two-screwed, three-screwed, or four-screwed internal fixator was combined with sacroiliac screws as the fixation system, respectively. Group D used a steel plate combined with sacroiliac screws as the fixation system. All Tile C3 pelvic fracture specimens were created by the same method (dislocation of bilateral sacroiliac joints and rupture of bilateral symphysis pubis) [5]. Rupture of the symphysis pubis was performed by sawing off the superior and inferior pubic ramus at a distance of 3–5 cm from the pubic symphysis center, and dislocation of the sacroiliac joints was performed by cutting off all the sacroiliac ligaments sharply (Fig. 2).

### Fixation of specimens

**Fig. 3:**
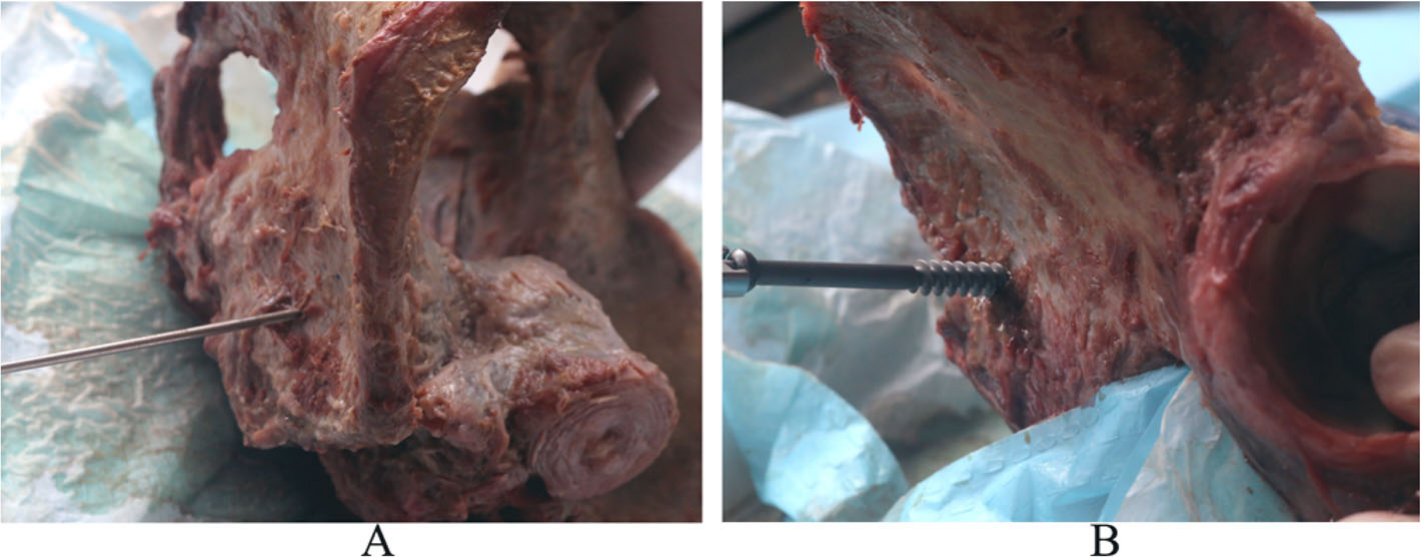
Insertion of sacroiliac screw. **a** Needle placement. **b** Screw placement

The sacroiliac joints of each specimen were stabilized with 7.3 mm hollow sacroiliac screws (Shandong Wei Gao Company, Weihai, China) using the same method, as described below. After reduction, the sacroiliac joints were fixed with a reduction clamp. The insertion positions on both sides were at the posterior 1/3 intersection of the anterior superior iliac spine and the posterior superior iliac spine. A 2.5 mm diameter guide needle was inserted with the needle tail sloping at an angle of approximately 10° upward and 20° backward into the middle anterior side of the ilium and the sacroiliac joints toward the sacral 1 vertebral body, using an electric drill. After confirming the position of the guide needle, the specimens were drilled using a hollow core bit. After measuring the depth, 7.3 mm sacroiliac screws of an appropriate length (65–90 mm) were inserted for stabilization (Fig. 3).

**Fig. 4:**
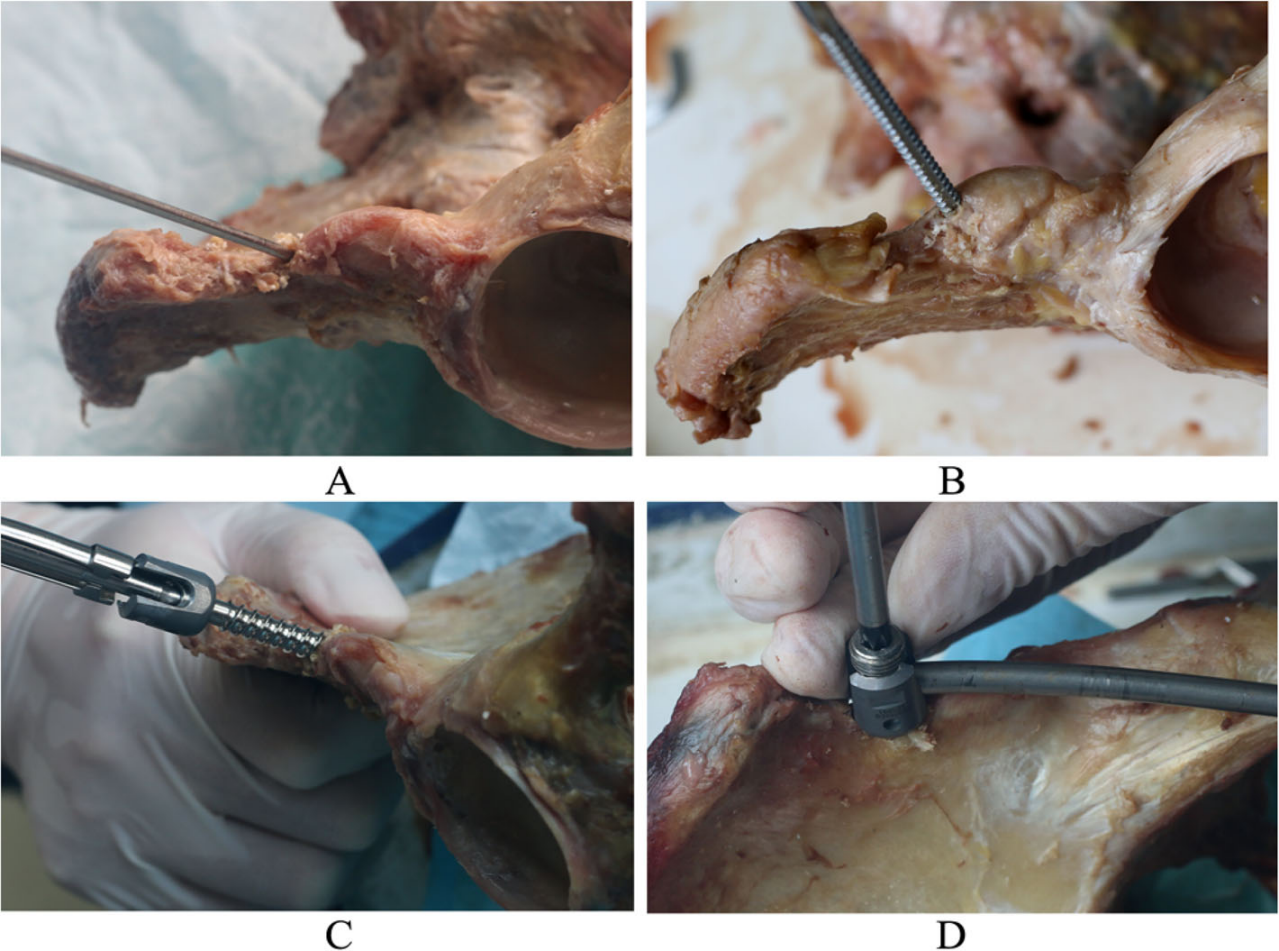
Insertion of anterior internal system screw. **a** Needle placement. **b** Trepanning, **c** Screw placement. **d** Fixing connection rod

Reduction of the anterior ring pubic ramus fractures was achieved with fixation clamps. Group A was stabilized using a two-screwed internal fixator system. The 2.5 mm diameter guide needles were inserted on both sides of the anterior ilium in the upper part of the inferior iliac spine, at a position about 5 mm toward the superior iliac spine. The angles between the needle and the horizontal plane, the sagittal plane and the coronal plane were approximately 55°, 26°, and 64°, respectively. After confirming positions of the guide needle, screw canals were created at the inner and outer plates of the ilium, and the depths were measured in order. One 7.5-mm-diameter and 65–90-mm-length titanium alloy universal anterior pelvic ring screw (Xiamen Da Bo Company, Fujian, China) was inserted into each canal following the guide needle position. The end of the screws protruded from the bone by 10–15 mm. The pre-shaped 6-mm-diameter titanium rods forming a forward bow were cut to the desired length and stabilized using two screws and fixed to the specimens under pressure (Fig. 4).

**Fig. 5:**
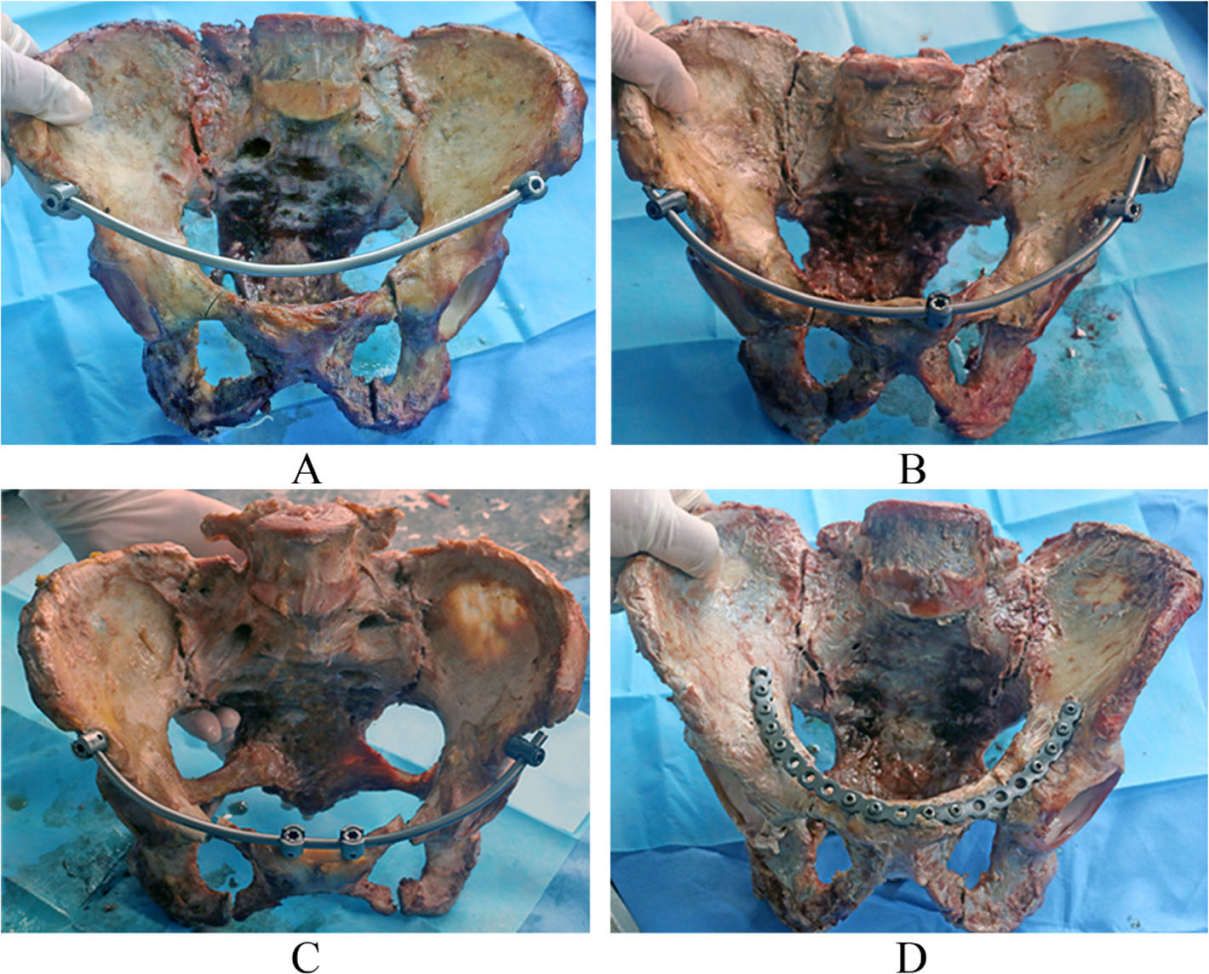
Fixation models of facture specimens. **a** Fixation model 1. **b** Fixation model 2. **c** Fixation model 3. **d** Fixation model 4

The specimens in group B were stabilized by the same method at the anterior inferior iliac spine using universal anterior pelvic ring screws. One 4.5-mm-diameter and 35–45-mm-length universal anterior ring fixation screw was inserted at the right side of the pubis 1 cm away from the pubis symphysis midline following the guide needle. The pre-shaped 6 mm diameter titanium rods forming a forward bow were cut to the appropriate length, stabilized with three screws, and fixed to the specimens under pressure (Fig. 5).

The specimens in group C were stabilized using the same techniques at the anterior inferior iliac spine using universal anterior pelvic ring screws. Two 4.5-mm-diameter, 35–45-mm-length universal anterior pelvic ring screws were inserted at both sides of the pubis 1 cm away from the pubis symphysis midline following the guide needle. Accordingly, the pre-shaped 6 mm diameter titanium rods forming a forward bow were cut to the desired length, stabilized with four screws and fixed to the specimens under pressure (Fig. 5).

In group D, following reduction of the anterior pelvic ring fracture, a pre-shaped reconstruction locking plate and screws were used for stabilization (Fig. 5).

**Fig. 6:**
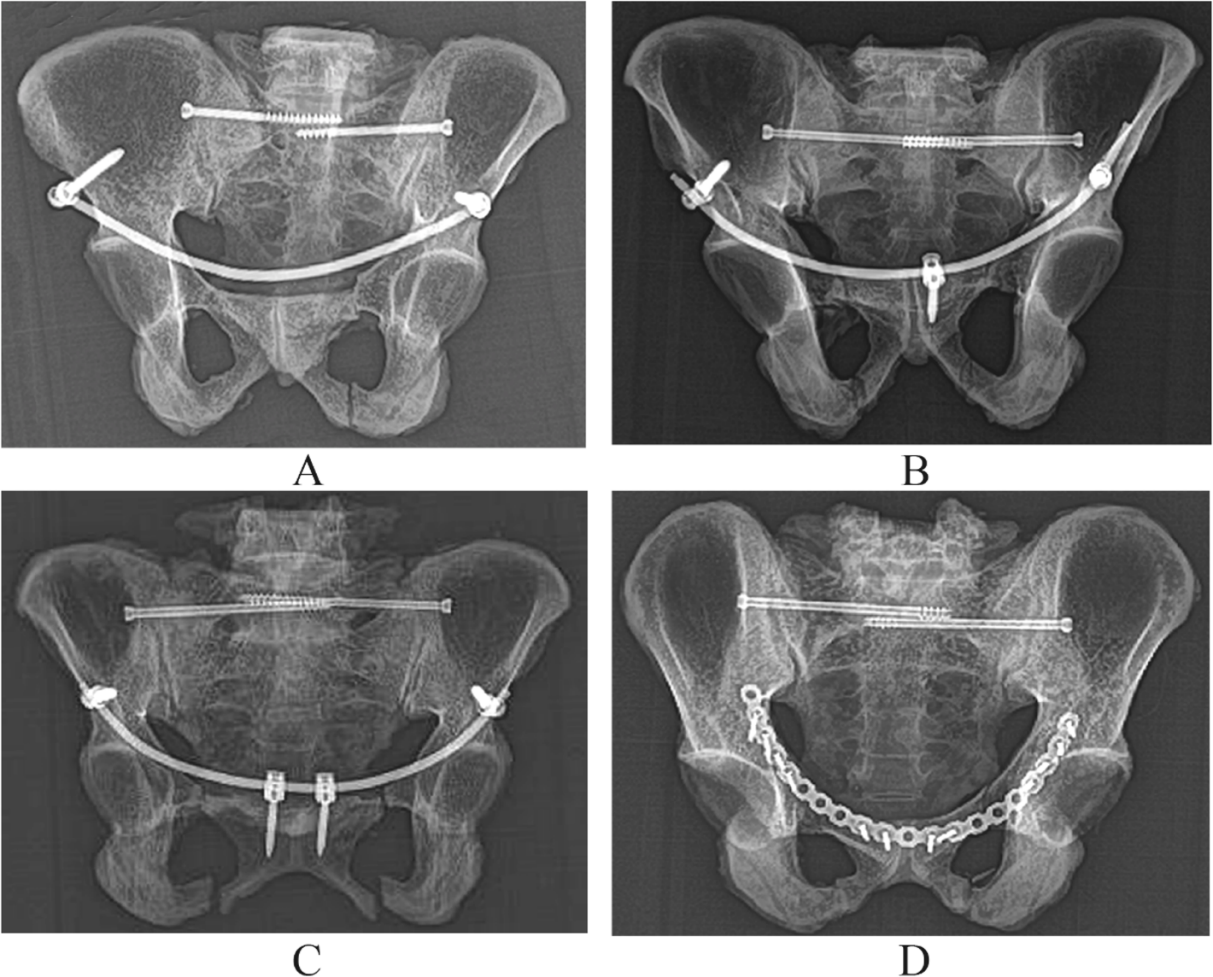
CT images of fixation models. **a** Fixation model 1. **b** Fixation model 2. **c** Fixation model 3. **d** Fixation model 4

**Fig. 7:**
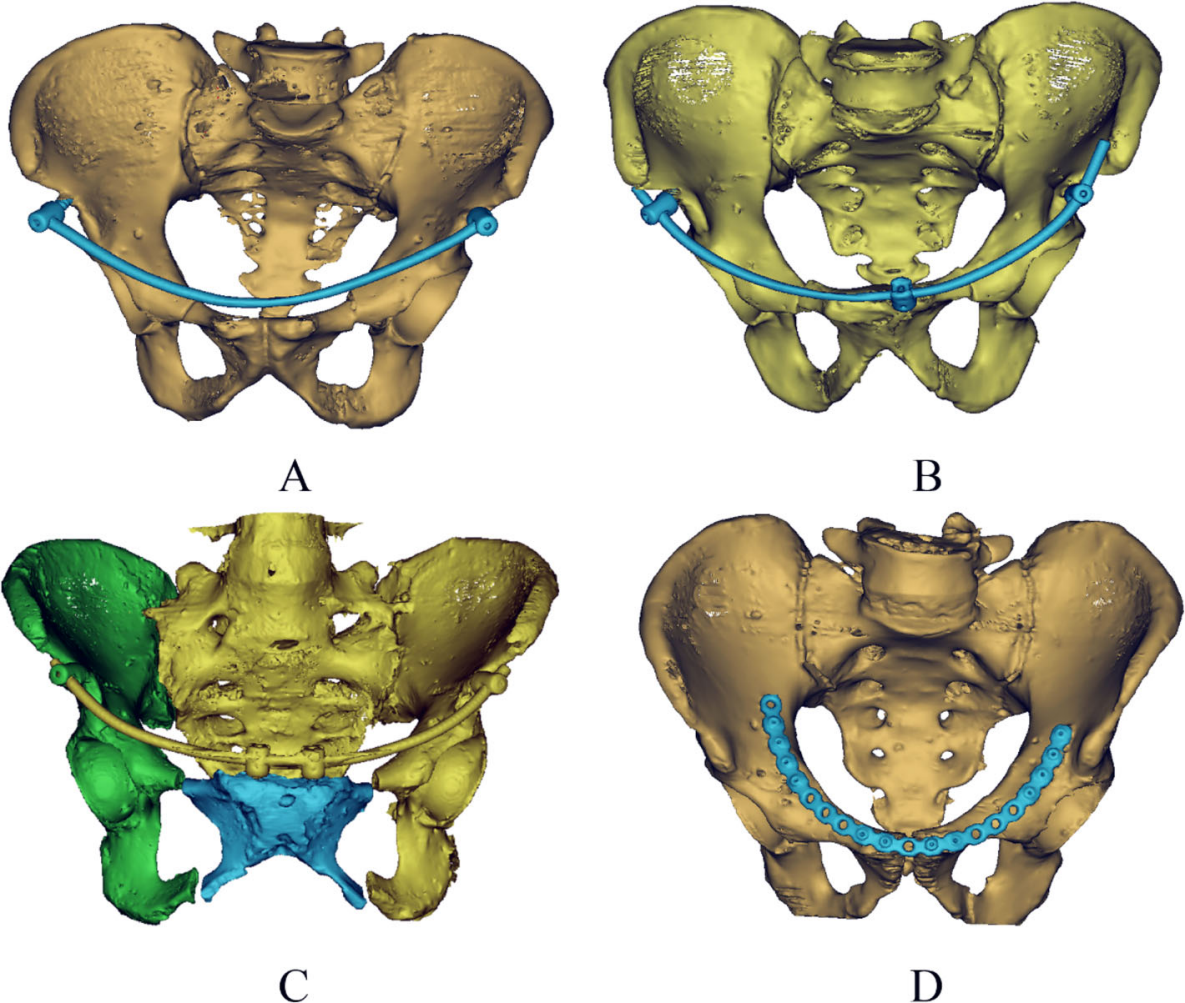
3D CT images of fixation models. **a** Fixation model 1. **b** Fixation model 2. **c** Fixation model 3. **d** Fixation model 4

After fixation, all the fracture specimens were observed by X-ray and CT scanning (In situm CT, SINOVISION Technologies (Beijing) Co., Ltd, Beijing, China). Reduction of fracture specimens, fixation positions, and length were confirmed (Figs. 6 and 7).

### Biomechanical measurement methods

**Fig. 8:**
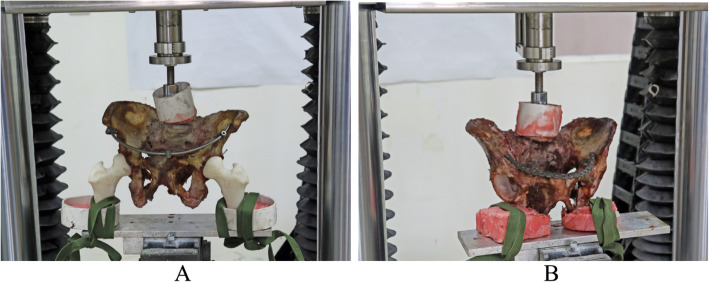
Biomechanical testing. **a** Standing posture. **b** Sitting posture

All the specimens were reconstructed based on CT scanning data of the one third middle upper portion of the bilateral femurs using a 3D printer (Dimension® BST/SST 1200es 3D Printer, Stratasys Inc., Eden Prairie, MN, USA). The printed femur matched-up with the specimens. The L5 vertebral body and the distal femur in the pelvic specimen were cast and embedded with resin denture powder using a 5-mm denture base. All specimens were initially tested in the standing position. Prior to the test, to measure the displacement, two 1.5-mm Kirschner wires were inserted at both sides of the bilateral sacroiliac joints and the superior pubic ramus fractures approximately 1 cm distant from the sacroiliac joints or the pubic fracture. The end of the needle was exposed at the outside of the cortex by approximately 1 cm. All the specimens were placed on the biomechanical testing platform of a Bose ElectroForce 3510 high precision biomaterials experimental system (Bose Corporation, Eden Prairie, MN, USA). The specimens simulated the normal physiological standing position followed by fixation (Fig. 8). Three on-and-off preprocessing loads were applied between 0 and 400 N with a rate of increase of 20 N/s. An electronic digital micrometer (Jinhua Shijiang Tools Co. Ltd., Jinhua, China; precision: 0.001 mm) measured the distance between the original positions of the two Kirschner wires three times, and the mean value was calculated. Gradient vertical loads of 100, 200, 300, 400, 500, and 600 N (increasing at a rate of 20 N/s) were applied by the biomechanical testing machine. After reaching the required pressure loads, the force was maintained for 30 s and then the distance between the positions of the Kirschner wires was measured three times, and the mean value was calculated. After testing all the specimens in a standing posture, the femurs were removed. The ischial tuberosity was fixed by embedding using the resin denture powder platform with a 5 mm denture base. The specimens then simulated a normal physiological sitting posture and were stabilized on the biomechanical testing platform (Fig. 8). The specimens in the sitting posture then underwent biomechanical testing as described for those in the standing posture.

### Statistical analysis

Statistical analysis was performed using SPSS version 19.0 software (IBM SPSS Statistics for Windows, Armonk, NY, USA). Data are presented as X ± s. The independent *t*-test was used to compare the data within the group and one-way analysis of variance (ANOVA) was used for data comparison between groups. Levene’s test was applied for homogeneity testing of variance, LSD test was used to analyze the equal variance of data, and Dunnett’s T3 method was used for testing data with heterogeneity of variance. A *P* value < 0.05 indicated a statistically significant difference.

## Results

### General observations

The stability of all specimens was good and no obvious pelvic tilt was observed. The loading platform of the L5 vertebral body, femur, and ischium were in close contact with the biomechanical testing machine. Pelvic specimens did not show any significant deformation, any new onset fractures, any fractures of the femoral prosthesis, or any rupture, pulling out, or loss of internal fixators. The denture powder platform showed no deformation.

### Displacements of sacroiliac joints and shifts in the gap of pubis ruptures on pelvic specimens

Displacements of sacroiliac joints and shifts in the gap of pubis ruptures are detailed in Tables 1, 2, 3, and 4.

**Table 1:** Displacements of sacroiliac joints in standing posture (X ± S, *n* = 4, mm)

Loads(*N*)	Group A		Group B		Group C		Group D	
	Left	Right	Left	Right	Left	Right	Left	Right
200	0.8400±0.00816	0.8400±0.00816	0.8350±0.01291	0.8400±0.08160	0.6850±0.01000	0.6825±0.00500	0.7850±0.01291	0.7850±0.00577
400	1.9500±0.00816	1.9550±0.01291	1.9575±0.00957	1.9575±0.00500	1.5325±0.01258	1.5300±0.01155	1.9300±0.00816	1.9300±0.00816
600	3.2150±0.00577	3.2175±0.00500	3.2200±0.01414	3.2225±0.00957	2.9700±0.00816	2.9725±0.00957	3.0775±0.00957	3.0775±0.00500

Every two groups were compared by the independent *t*-test, *P* > 0.05

**Table 2:** Shifts in the gap of pubis ruptures in the standing posture (X ± S, *n* = 4, mm)

Loads(*N*)	Group A		Group B		Group C		Group	
	Left	Right	Left	Right	Left	Right	Left	Right
200	0.8750±0.00577	0.8775±0.00500	0.7300±0.01155	0.7325±0.01258	0.7900±0.00816	0.7900±0.01414	0.6825±0.01500	0.6825±0.00500
400	1.6400±0.00816	1.6375±0.00500	1.6250±0.00577	1.6325±0.00957	1.9350±0.00577	1.9275±0.00500	1.5275±0.00500	1.5350±0.00577
600	3.2550±0.01000	3.2575±0.00500	3.1450±0.01732	3.1475±0.02500	3.0700±0.00816	3.0750±0.01291	2.9725±0.00500	2.9725±0.00957

Every two groups were compared by the independent sample *t*-test, *P* > 0.05

**Table 3:** Displacements of sacroiliac joints in the sitting posture (X ± S, *n* = 4, mm)

Loads (*N*)	Group A		Group B		Group C		Group D	
	Left	Right	Left	Right	Left	Right	Left	Right
200	0.2800±0.00816	0.2700±0.00816	0.2600±0.00816	0.2625±0.00957	0.2475±0.00500	0.2500±0.01155	0.2475±0.00500	0.2450±0.00577
400	0.4650±0.00577	0.4750±0.00577	0.4475±0.00957	0.4475±0.00500	0.4175±0.01708	0.4225±0.00957	0.4175±0.01500	0.4225±0.12580
600	0.6650±0.00577	0.6675±0.00500	0.6525±0.00957	0.6525±0.00500	0.6300±0.00816	0.6300±0.00816	0.6325±0.00957	0.6300±0.00816

Every two groups were compared by the independent *t*-test, *P* > 0.05

**Table 4:** Shifts in the gap of pubis ruptures in the sitting posture (X ± S, *n* = 4, mm)

Loads (*N*)	Group A		Group B		Group C		Group D	
	Left	Right	Left	Right	Left	Right	Left	Right
200	0.3675±0.00957	0.3675±0.00500	0.3450±0.01000	0.3525±0.00500	0.3400±0.00816	0.3375±0.01258	0.3350±0.00577	0.3350±0.00577
400	0.5450±0.00577	0.5450±0.00577	0.4900±0.00816	0.4900±0.00816	0.4600±0.00816	0.4600±0.00816	0.4650±0.01291	0.4650±0.00577
600	0.7725±0.00500	0.7550±0.03697	0.7400±0.00816	0.7450±0.01291	0.7150±0.00577	0.7075±0.00500	0.7100±0.01414	0.7125±0.00957

Every two groups were compared by the independent *t*-test, *P* > 0.05

**Fig. 9:**
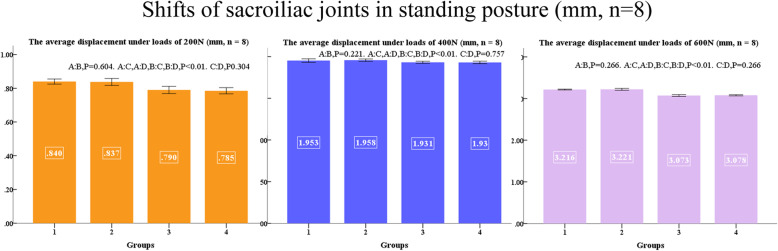
Shifts of sacroiliac joints in standing posture (mm, *n* = 8)

**Fig. 10:**
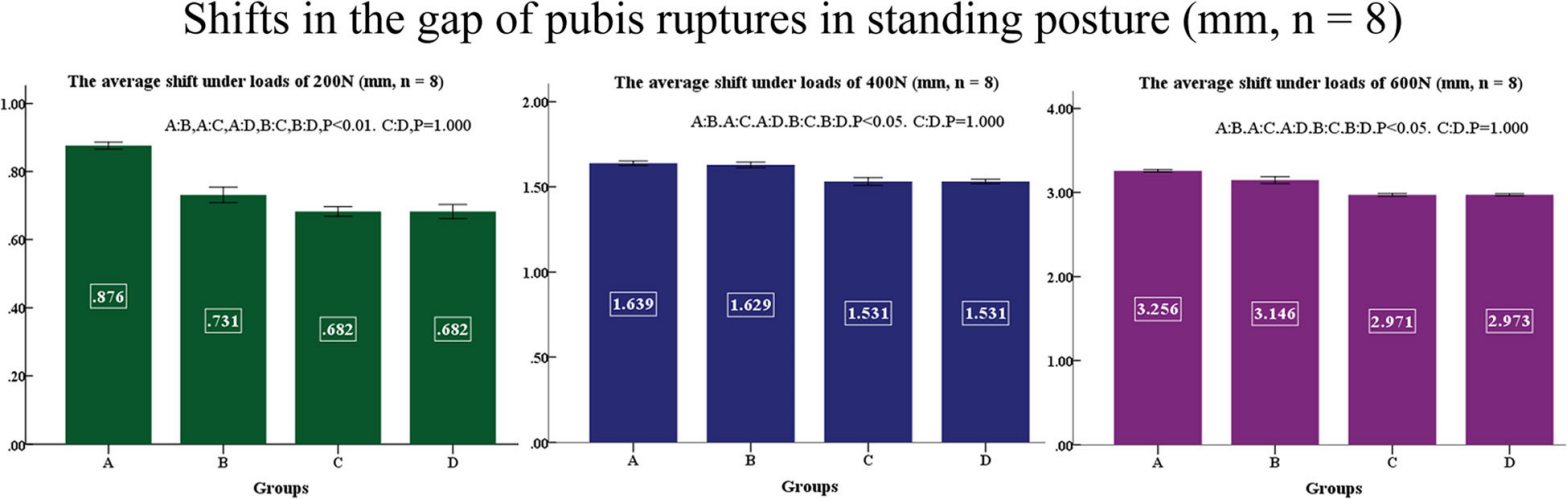
Shifts in the gap of pubis ruptures in standing posture (mm, *n* = 8)

**Fig. 11:**
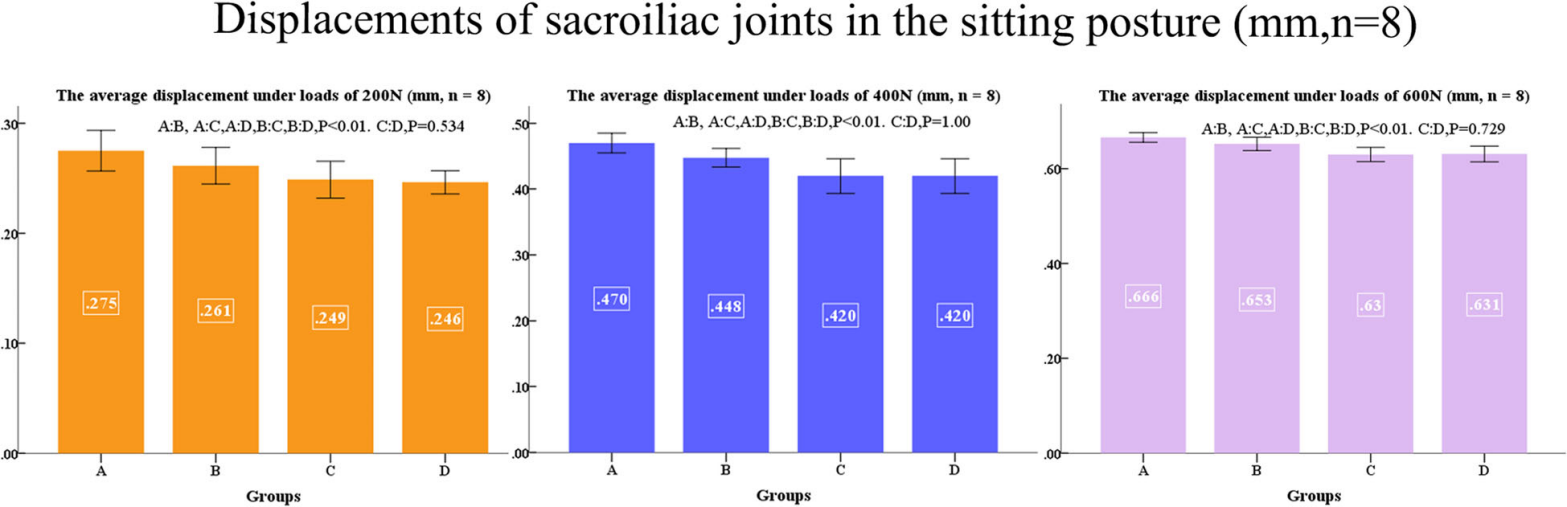
Displacements of sacroiliac joints in the sitting posture (mm, *n* = 8)

**Fig. 12:**
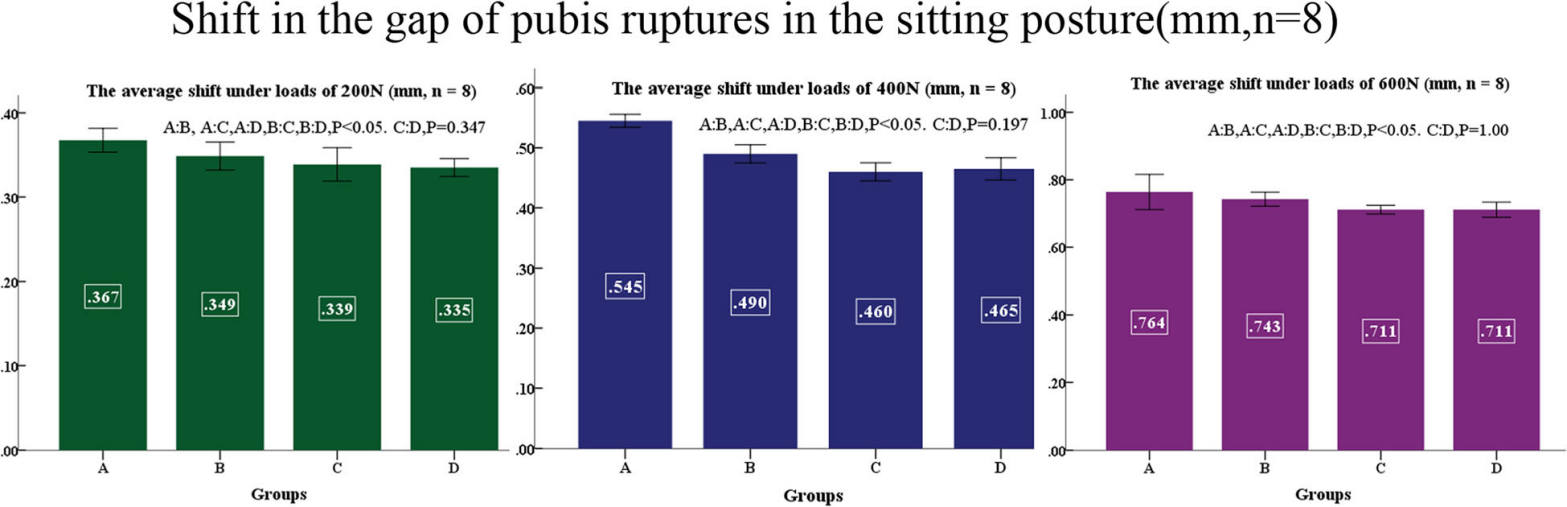
Shifts in the gap of pubis ruptures in the sitting posture (mm, *n* = 8)

No difference was found between left and right, displacements of sacroiliac joints and shifts of pubis ruptures in the standing posture at applied loads of 200, 400, and 600 N are shown in Figs. 9 and 10. Displacements of sacroiliac joints and shifts of pubis ruptures in the sitting posture at applied loads of 200, 400, and 600 N are shown in Figs. 11 and 12.

## Discussion and conclusions

With the development of minimally-invasive techniques, sacroiliac screws and pelvic anterior ring internal fixation systems have been widely applied in the clinical field and exhibited good results. To date, the internal fixation system has been primarily used in treatment of Tile B pelvic fractures. However, some clinicians have gradually begun to apply an internal fixation system in cases of Tile C1 pelvic fracture [6]. Tile C3 pelvic fractures are one type of extremely unstable pelvic fracture which are caused by high energy injuries. These fractures usually occur in conjunction with injuries of other chest and abdominal organs and fractures of other bones. In early treatment, lift support and damage control should be the priority. To achieve better functional recovery of patients, minimally-invasive internal fixation treatment should be chosen for early or late pelvic fracture surgery. Whether the use of such minimally-invasive techniques using an internal fixator combined with sacroiliac screws is an effective fixation method for Tile C3 pelvic fractures has not yet been investigated in a biomechanical study. Performing mechanical testing of pelvic specimens in the traditional way can simulate the displacement and damage to the body [7]. In this article, Tile C3 pelvic fractures were created in fresh pelvic specimens and the major ligaments were retained to simulate the situation close to real pelvic fractures. Biomechanical properties were compared among fixation systems of Tile C3 pelvic fractures using a two-screwed, three-screwed, or four-screwed anterior internal fixator or a steel plate combined with sacroiliac screws.

Reduced pelvic shifts indicate greater stability [8]. After long-term follow-up of pelvic fractures in postoperative patients, Tornetta and Matta and Dujardin et al. proved that patients had better prognosis when shifts of pelvic fractures were less than 1 cm [9, 10]. The results of our study showed that the shifts of the posterior pelvic ring joints and the anterior pelvic ring fractures in the standing posture were less than 3.3 mm with all fixation systems. Shifts of those fixation systems in the sitting posture were less than 1 mm. The findings revealed that all fixation methods can provide biomechanical stability for Tile C3 pelvic fractures. Under loads of 200, 400, and 600 N in our study, the displacement of sacroiliac joints and pubic fractures in every specimen showed similar changes. In both standing and sitting postures, shifts in groups A and B were greater than those in groups C and D, and significant differences were shown in the comparison. However, there was no significant difference when comparing group C to group D. These findings revealed that groups C and D exhibited similar biomechanical stability and both had better biomechanical stability than groups A and B. With all the fixation models, when the shifts of pubis fractures of the anterior ring increased, the shifts of the sacroiliac screws in the posterior ring also increased. This proved that when the stability of the anterior pelvic ring increased, the stability of the posterior pelvic ring would increase correspondingly.

No relevant articles have yet reported whether increasing the number of screws in the pubic area can provide better stability of an anterior ring internal fixation system. In this study, under loads of 200, 400, and 600 N, the results showed that the shifts with the different fixation models of anterior ring pubic ramus fractures declined gradually in both standing posture and inferior sitting posture from group A to group C. Significant statistical differences were shown across the groups. As the number of screws in the pubic area increased, the biomechanical stability of the internal fixation system increased. Comparing group C and group D, they showed similar biomechanical stability; however, group C fixation was obviously more convenient to perform than group D.

Letournel was the first to suggest the use of sacroiliac screws to fix pelvic fractures in 1978. Subsequently this method was applied and promoted by Matta and Sauced, and it has proved to be an effective technique. Over the following three decades, many scholars have studied this method in depth, both via research and in clinical use. The percutaneous sacroiliac screw is one type of central internal fixation method passing through the broken end of the fracture. Previous biomechanical studies [11] proved that internal fixation with percutaneous iliac screws provided adequate stability and was effectively able to resist vertical shear and torsion forces. In 2004, Sagi’s group [12] demonstrated that when the pubic symphysis was fixed, one screw was adequate to stabilize the fractures, and there was no requirement for a second screw. All the fracture specimens in this study had dislocated bilateral sacroiliac joints, and the sacroiliac joints were fixed with one sacroiliac screw. Under loads of 600 N, shifts of the sacroiliac joints in the standing posture were within 3.0–3.3 mm, while shifts in the sitting posture were less than 1 mm. In both cases, sacroiliac screw fixation provided stability of the posterior pelvic ring. The efficacy of sacroiliac screw fixation depended on the accuracy of screw insertion [13]. Improper insertion of screws can easily cause iatrogenic injury to sacral nerves and anterior sacral vessels [14]. Recently, the safety of internal fixation with percutaneous sacroiliac screws has been greatly improved using computer-assisted navigation systems. The operation time has been shortened and the time of exposure to X-ray irradiation during surgery has been reduced [15]. Fixation of the sacroiliac joints with percutaneous sacroiliac screws can thus provide adequate biomechanical stability. This method was considered to be the preferred method of posterior pelvic fracture fixation, because it exhibited numerous advantages such as minimal trauma, little bleeding, a shortened operation time, and rapid fracture healing [16, 17].

Use of a steel plate with screw fixation or front channel screw fixation systems are the traditional treatments for anterior pelvic fractures. Steel plate with screw fixation has a good effect; however, it exhibits some disadvantages such as greater injury caused by the procedure and long operation time. Front channel screw fixation requires a long period of technical study and doctors with high technological skills. Furthermore, this method is not suitable for splintered anterior pelvic ring fractures. In 2009, Kutter et al. applied rod systems for percutaneous anterior pelvic ring fixation in unstable pelvic fractures, a technique known as anterior subcutaneous pelvic internal fixation (ASPIF) [18]. This technique has gradually been adopted in clinical practice due to its advantages such as minimal trauma and greater convenience. This method has also been termed the anterior subcutaneous internal fixator (INFIX) method by Vaidya since 2011 [19]. Many researchers have studied the anatomical and clinical aspects of internal fixation systems [20–22]. Their results revealed that the safety of such internal fixation systems was reliable and showed good effects. Our research results show that the INFIX system provided biomechanical stability of anterior pelvic ring fractures. It has been applied for minimally-invasive treatment of anterior ring fractures.

Following biomechanical testing, Vigdorchik et al. [23] stated that anterior pelvic ring plate fixation showed greater stability than either the screw–rod system or external fixation. In addition, the screw–rod system was stiffer than external fixation. In our study, shifts of all anterior pelvic ring fractures in the standing posture were less than 3.3 mm and shifts of anterior pelvic fractures in the sitting posture were all less than 0.8 mm. All fixation methods provided biomechanical stability in fixing anterior ring fractures. Shifts of anterior ring fracture specimens which were fixed using two-screwed or three-screwed internal fixation systems were greater than those fixed with a steel plate with screws, and these differences were statistically significant. In contrast, shifts of fractures fixed with a four-screwed internal system showed no difference compared to those fixed with steel plates with screws. These findings confirmed that anterior ring fixation using a steel plate with screws was more stable than the use of a two-screwed or three-screwed internal fixation system and that a four-screwed internal fixation system was as stable as a fixation system using a steel plate with screws.

## Limitations

Because it was difficult to obtain fresh pelvic specimens, the number of specimens was limited. The original femoral tissues of pelvic specimens in this study were not retained, therefore femurs were created by 3D printer instead; furthermore, the ligaments of the hip joints were missing. Because there were differences in comparison with the real situation, this may have influenced the results.

## Conclusion

An anterior ring fixation system combined with sacroiliac screws effectively stabilized Tile C3 pelvic fractures. As the number of anterior fixation screws increased, the biomechanical stability increased correspondingly.

## Future study

Our further studies will focus on clinical application of Tile C pelvic fracture treatment using an internal fixator system combined with sacroiliac screws. Their clinical effects will be investigated.

## Data Availability

The datasets used and analyzed in the study are available on request to the corresponding author.

## References

[1] Cole PA, Gauger EM, Anavian J, Ly TV, Morgan RA, Heddings AA (2012). Anterior pelvic external fixator versus subcutaneous internal fixator in the treatment of anterior ring pelvic fractures. J Orthop Trauma..

[2] Hiesterman TG, Hill BW, Cole PA (2012). Surgical technique: a percutaneous method of subcutaneous fixation for the anterior pelvic ring: the pelvic bridge. Clin Orthop Relat Res..

[3] Vaidya R, Colen R, Vigdorchik J, Tonnos F, Sethi A (2012). Treatment of unstable pelvic ring injuries with an internal anterior fixator and posterior fixation: initial clinical series. J Orthop Trauma..

[4] Gardner MJ, Mehta S, Mirza A, Ricci WM (2012). Anterior pelvic reduction and fixation using a subcutaneous internal fixator. J Orthop Trauma..

[5] Tao W, Wei C, Qi Z (2015). Biomechanical comparison of two kinds of internal fixation in a type C zone II pelvic fracture model. Chin Med J..

[6] Vaidya R, Tonnos F (2016). The anterior subcutaneous pelvic fixator (INFIX) in an anterior posterior compression type 3 pelvic fracture. J Orthop Trauma.

[7] Garcia JM, Doblar EM, Sera B (2000). Three dimensional finite element analyses of several internal and external pelvis fixations. J Biomech Eng.

[8] Lee CH, Hsu CC, Huang PY (2017). Biomechanical study of different fixation techniques for the treatment of sacroiliac joint injuries using finite element analyses and biomechanical test. Comput Biol Med..

[9] Tornetta P, Matta JM (1996). Outcome of operatively treated unstable posterior pelvic ring disruptions. Clin Orthop Relat Res.

[10] Dujardin FH, Hossenbaccus M, Duparc F, Biga N, Thomine JM (1998). Long-term functional prognosis of posterior injuries in high-energy pelvic disruption. J Orthop Trauma.

[11] Kim JJ, Jung CY, Eastman JG (2016). Measurement of optimal insertion angle for iliosacral Screw fixation using three dimensional computed tomography scans. Clin Orthop Surg.

[12] Sagi HC, Ordway NR, DiPasquale T (2004). Biomechanical analysis of fixation for vertically unstable sacroiliac dislocations with iliosacral screws and symphyseal plating. J Orthop Trauma..

[13] Salari P, Moed BR, Bledsoe JC (2015). Supplemental S1 fixation for type C pelvic ring injuries:biomechanical study of a long iliosacral versus a transsacral screw. J Orthop Trauma.

[14] Takao M, Nishii T, Sakai T (2014). Iliosacral screw insertion using CT-3D-fluoroscopy matching navigation. Injury.

[15] Matityahu A, Kahler D, Krettek C, Stöckle U, Grutzner PA, Messmer P, Ljungqvist J, Gebhard F (2014). Three-dimensional navigation is more accurate than two-dimensional navigation or conventional fluoroscopy for percutaneous sacroiliac screw fixation in the dysmorphicsacrum: a randomized multicenter study. J Orthop Trauma.

[16] Peng KT, Li Y-Y, Hsu W-H (2013). Intraoperative computed tomography with integrated navigation in percutaneous iliosacral screwing. Injury.

[17] Kim JW, OH CW, OH JK, et al. (2013). Percutaneous iliosacral screwing in pelvic ring injury using three-dimensional fluoroscopy. J Orthop Sci.

[18] Kuttner M, Klaiber A, Lorenz T, Füchtmeier B, Neugebauer R (2009). The pelvic subcutaneous cross-over internal fixator [J]. Unfallchirurg.

[19] Vaidya R, Kubiak EN, Bergin PF, Dombroski DG, Critchlow RJ, Sethi A, Starr AJ (2012). Complications of anterior subeutaneous internal fixation for unstable pelvis fractures: a multicenter study. Clin Orthop Relat Res.

[20] Apivatthakakul T, Rujiwattanapong N (2016). “Anterior subcutaneous pelvic internal fixator (INFIX), Is it safe?”A cadaveric study. Injury..

[21] Bi C, Wang Q, Nagelli C, Wu J, Wang Q, Wang J (2016). Treatment of unstable posterior pelvic ring fracture with pedicle screw-rod fixator versus locking compression plate: a comparative study. Med Sci Monit..

[22] Rahul Vaidya, Adam Jonathan Martin, MatthewRoth, et al. Midterm radiographic and functional outcomes of the anterior subcutaneous internal pelvic fixator (INFIX) for pelvic ring injuries. J Orthop Trauma. 2017;31(5):252–9. 10.1097/BOT.0000000000000781 PMC5402711 28079731

[23] Vigdorchik JM, Esquivel AO, Jin X, Yang KH, Onwudiwe NA, Vaidya R (2012). Biomechanical stability of a supra-acetabular pedicle screw internal fixation device (INFIX) vs external fixation and plates for vertically unstable pelvic fractures. J Orthop Surg Res..

